# Barriers and facilitators to chemotherapy initiation and adherence for patients with HIV-associated Kaposi’s sarcoma in Kenya: a qualitative study

**DOI:** 10.1186/s13027-022-00444-0

**Published:** 2022-07-06

**Authors:** Devon E. McMahon, Rhea Singh, Linda Chemtai, Aggrey Semeere, Helen Byakwaga, Merridy Grant, Miriam Laker-Oketta, Celestine Lagat, Sigrid Collier, Toby Maurer, Jeffrey Martin, Ingrid V. Bassett, Lisa Butler, Samson Kiprono, Naftali Busakhala, Esther E. Freeman

**Affiliations:** 1 Department of Dermatology, Harvard Medical School, Massachusetts General Hospital, 50 Staniford St, Boston, MA 02114 USA; 2 Virginia Commonwealth University School of Medicine, Richmond, VA USA; 3 AMPATH, Moi University, Eldoret, Kenya; 4 Infectious Disease Institute, Kampala, Uganda; 5 University of KwaZulu-Natal, Durban, South Africa; 6 University of Washington, Seattle, WA USA; 7 Indiana University, Indianapolis, IN USA; 8 University of California San Francisco, San Francisco, CA USA; 9 University of Connecticut, Storrs, CT USA; 10 Department of Pharmacology and Toxicology, School of Medicine, College of Health Sciences, Moi University, Eldoret, Kenya

**Keywords:** Kaposi sarcoma, HIV/AIDS, Chemotherapy, Treatment, Adherence, Africa South of the Sahara, Kenya, Qualitative

## Abstract

**Background:**

Kaposi sarcoma is one of the most prevalent HIV-associated malignancies in sub-Saharan Africa and is often diagnosed at advanced stage of disease. Only 50% of KS patients who qualify for chemotherapy receive it and adherence is sub-optimal.

**Methods:**

57 patients > 18 years with newly diagnosed KS within the AMPATH clinic network in Western Kenya were purposively selected to participate in semi-structured interviews stratified by whether they had completed, partially completed, or not completed chemotherapy for advanced stage KS. We based the interview guide and coding framework on the situated Information, Motivation, Behavioral Skills (sIMB) framework, in which the core patient centered IMB constructs are situated into the socioecological context of receiving care.

**Results:**

Of the 57 participants, the median age was 37 (IQR 32–41) and the majority were male (68%). Notable barriers to chemotherapy initiation and adherence included lack of financial means, difficulty with convenience of appointments such as distance to facility, appointment times, long lines, limited appointments, intrapersonal barriers such as fear or hopelessness, and lack of proper or sufficient information about chemotherapy. Factors that facilitated chemotherapy initiation and adherence included health literacy, motivation to treat symptoms, improvement on chemotherapy, prioritization of self-care, resilience while experiencing side effects, ability to carry out behavioral skills, obtaining national health insurance, and free chemotherapy.

**Conclusion:**

Our findings about the barriers and facilitators to chemotherapy initiation and adherence for KS in Western Kenya support further work that promotes public health campaigns with reliable cancer and chemotherapy information, improves education about the chemotherapy process and side effects, increases oncology service ability, supports enrollment in national health insurance, and increases incorporation of chronic disease care into existing HIV treatment networks.

## Background

Kaposi sarcoma (KS) is one of the most common HIV-associated malignancies in sub-Saharan Africa (SSA) [1]. Despite significant progress in uptake of antiretroviral therapy (ART), most KS patients are diagnosed at an advanced stage of disease, requiring chemotherapy for treatment [1–4]. For patients with advanced KS, the combination of ART and chemotherapy improves KS complete response rates by approximately 20–40% as compared to ART alone [5–7]. However, up to 50% of advanced stage KS patients do not receive chemotherapy and adherence for those on chemotherapy remains sub-optimal [8, 9].

Prior studies of other HIV-associated malignancies in SSA described lack of infrastructure and support, lack of in-service support, weak referral networks, lack of finances and transport, loss of follow-up, and poor communication between physician and patient as barriers to treatment initiation and adherence [2, 10]. However, there is scarce data on the barriers and facilitators to chemotherapy treatment specifically in patients with HIV-associated KS, which may pose different challenges than for other HIV-associated malignancies such as cervical cancer and lymphoma [10].

The objective of this qualitative study conducted with people living with HIV-associated KS in Kenya was to identify and understand barriers and facilitators to chemotherapy initiation and adherence.

## Methods

This qualitative study is nested within a larger longitudinal epidemiological study of newly diagnosed adults with HIV-associated KS in western Kenya, where participants were enrolled using rapid case ascertainment (RCA) methodology promptly after diagnosis [11]. Of this group of patients, we purposively sampled patients for in-depth semi-structured interviews.

### Study setting

This study was conducted at the Academic Model Providing Access to Healthcare (AMPATH), a network of HIV primary care clinics in western Kenya from March 2019 through September 2019. This network includes 60 different clinical sites; participants were recruited for interviews from the following seven AMPATH locations: Moi Teaching and Referral Hospital (MTRH), Chulaimbo, Busia, Kitale, Webuye, Mukhobola, and Usain Gisu District Hospital (UGDH). MTRH is the tertiary referral center for AMPATH, which provides oncology care.

### Participant selection

Eligible participants were HIV-positive, > 18 years, and had been enrolled in our parent study to rapidly identify cases of Kaposi's sarcoma [11]. Participants were recruited by purposive sampling to include patients who i) had not initiated chemotherapy after a period of > 30 days from when a provider recommended chemotherapy initiation, ii) who had initiated but not completed a standard chemotherapy course, and iii) completed chemotherapy. Potential participants were approached by the research team in person or by telephone. Those willing to participate were screened for inclusion criteria.

### Data collection

**Fig. 1:**
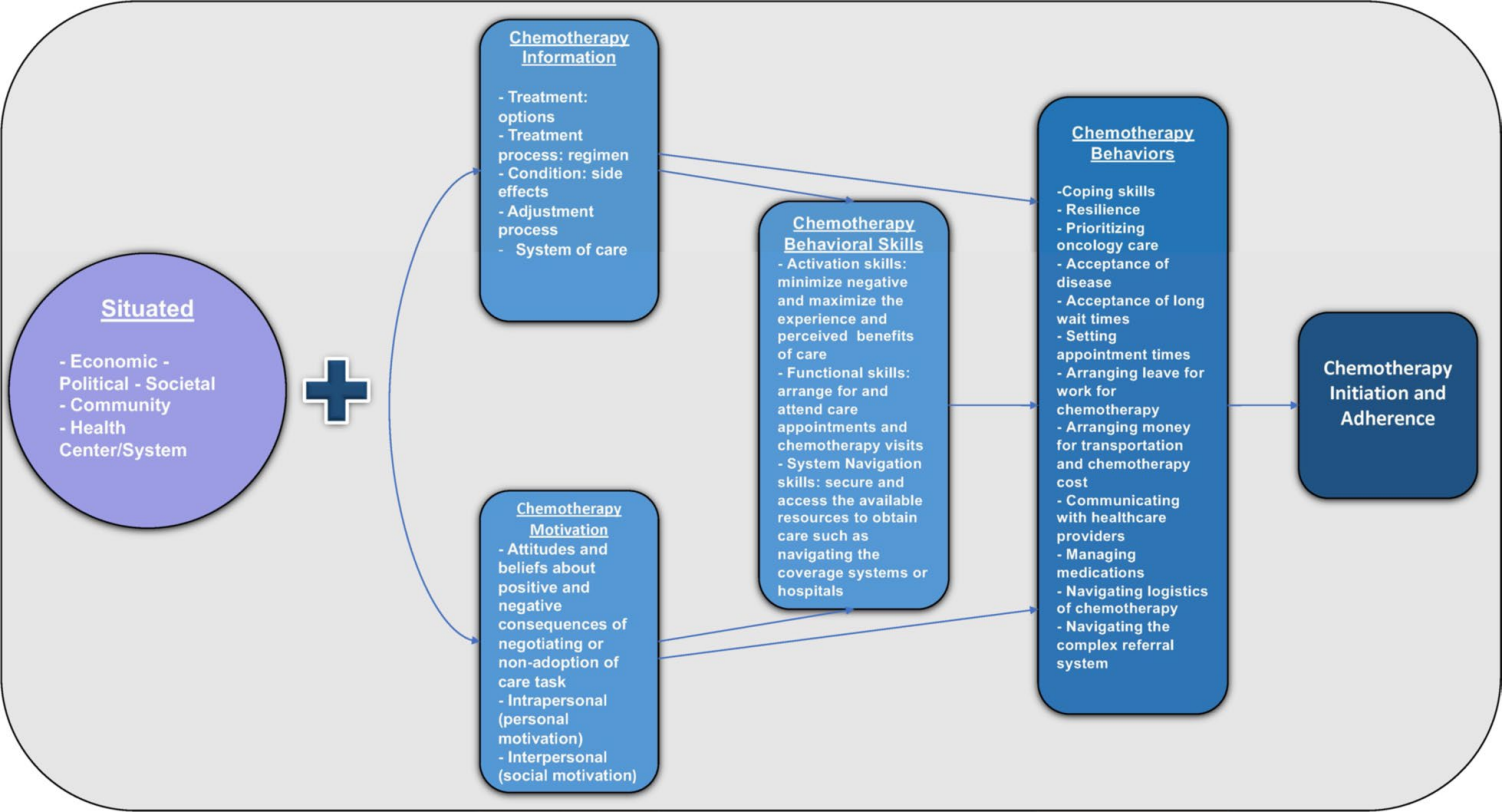
Proposed situated information motivation behavior model for initiation and adherence of chemotherapy [12]

The interview guide and initial coding framework were based off of the situated Information Motivation Behavior model (sIMB), developed by Amico et al. as a model for care initiation and maintenance for chronic medical conditions [12]. In this model, each of the core IMB model constructs of information, motivation, and behavioral skills are situated into the socioecological context of receiving care, which we adapted to our care setting and study question (Fig. 1).

Data was collected from March 2019 through September 2019. In a private setting, a Swahili and Kalenjin speaking researcher (LC) obtained verbal consent and conducted one-on-one, hour-long semi-structured interviews. The interview guides included knowledge and attitudes towards KS, cancer, and HIV, barriers and facilitators to chemotherapy initiation and completion, and possible interventions for treatment. Participants were reimbursed for travel. The Massachusetts General Hospital Institutional Review Board and the Kenyan Institutional Review Committee approved the protocol.

### Data analysis

Audio recordings were professionally transcribed and translated into English. Data were analyzed with NVivo using a framework approach. After each interview, authors LC and EF created a list of major themes generated during the interviews. These themes were used to develop an initial codebook. This codebook was then applied to 15 interviews by authors DM and MG. This initial coding structure was iteratively compared during weekly meetings, and discrepancies were resolved by senior author EF. Codes were compared until each had a Kappa score of greater than 0.6 to ensure high inter-rater reliability. Coding discrepancies were resolved through a group consensus. The remaining interviews were split in half and coded by DM and MG individually.

The approach to analysis combined deductive and inductive analysis in an iterative fashion. A deductive framework analysis approach was used during the initial phase of coding in order to assign interview themes to the IMB and socioecological model. Inductive analysis was also implemented to apply our research question within the Western Kenyan setting.

## Results

**Fig. 2:**
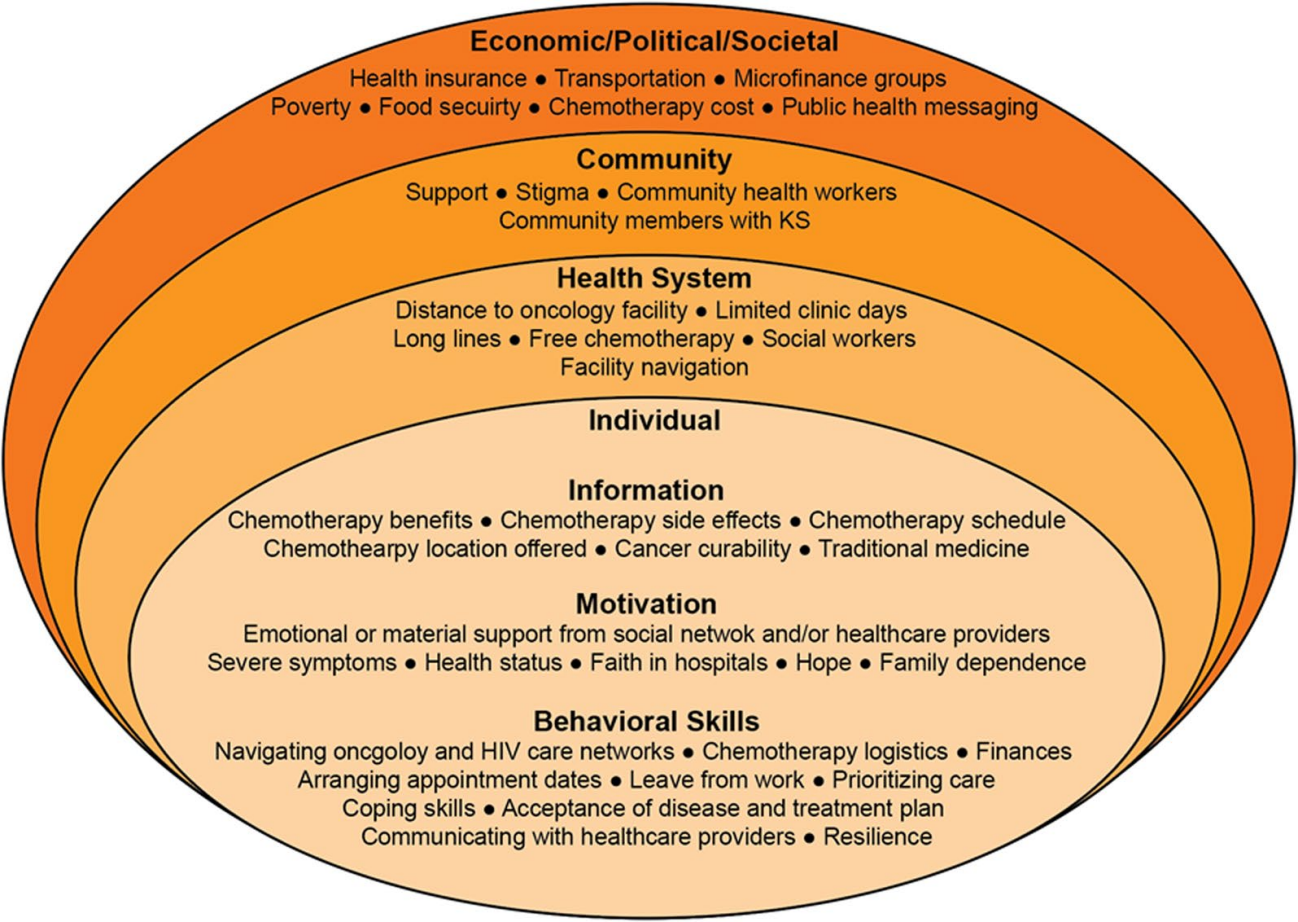
Major themes for initiation of and adherence to chemotherapy, structured within the Situated Information, Motivation, Behavior (sIMB) framework as developed by Amico et al. [12]

57 participants underwent semi-structured interviews. Participants were median age 37 (IQR 32–41), majority male (68%), and were interviewed at a variety of health centers and one referral hospital throughout Western Kenya (Table 1). Most themes within the sIMB model applied to both chemotherapy initiation and adherence, as described below and outlined in Table 2 and depicted in Fig. 2.

**Table 1:** Interview participant characteristics

Characteristic	Non-starter (n = 17)	Non-completer (n = 19)	Completer (n = 21)	Overall (n = 57)
Age, years (Median, IQR)	34 (29–39)	35 (31.5–41)	34 (32–43)	35 (32–41)
Male sex	7 (42.2%)	12 (63.2%)	20 (95.2%)	39 (68.4%)
**Health facility**				
Moi Teaching and Referral Hospital	6 (35.3%)	5 (26.3%)	2 (9.5%)	13 (22.8%)
AMPATH—Chulaimbo	2 (11.8%)	9 (47.4%)	14 (66.7%)	25 (43.9%)
AMPATH—Busia	4 (23.5%)	1 (5.3%)	4 (1.9%)	9 (15.8%)
AMPATH—Kitale	5 (29.4%)	1 (5.3%)	0 (0.0%)	6 (10.5%)
AMPATH—Webuye	0 (0.0%)	1 (5.3%)	1 (4.8%)	2 (3.51%)
AMPATH—Mukhobola	0 (0.0%)	1 (5.3%)	0 (0.0%)	1 (1.75%)
AMPATH—UGDH	0 (0.0%)	1 (5.3%)	0 (0.0%)	1 (1.75%)

**Table 2:** Themes of facilitators and barriers to Kaposi’s Sarcoma treatment adherence and completion, illustrated with representative quotes.

Themes	Quotes	Type of Theme	Incidence Density Number of quotes per theme
**Structural**—Policy			
Facilitators			
National Health Insurance Fund	“[The doctor] knows very well that I have the active NHIF card so he can do the medication without looking back so I really didn't suffer financially…” (#51, M, 43, Chulaimbo, treatment completer)	Both	**High ** Total: 72 Completer: 39 Non-Completer: 20 Non-Starter: 13
Public Health Messaging	“It [public radio messages] helped taking my medication…I heard them say if cancer is properly treated and if you follow through with what you are told by your doctor then it will resolve.” (#63, F, 35, Chulaimbo, treatment non-completer)	Both	**Low ** Total: 4 Completer: 1 Non-Completer: 1 Non-Starter: 2
Microfinance Groups	“I do small businesses and God helps me, because when I take the loan, I work hard, and I am able to pay off and take another loan.” (#36, M, 43, MTRH, treatment completer)	Both	**Medium ** Total: 81 Completer: 36 Non-Completer: 31 Non-Starter: 14
PEPFAR / AMPATH funding	“I appreciate this AMPATH, I do not know whether it is AMPATH because sometimes when I come here, they reimburse me transport and I find it very easy for me to come.” (#62, M, 35, Chulaimbo, treatment non-completer)	Both	**Low** Total: 24 Completer: 13 Non-Completer: 8 Non-Starter: 3
Barriers			
Lack of Affordable Public Transportation	“Like me when I come to Chulaimbo you know they… I used to come by vehicle when I was able to at least walk, but nowadays I have to hire motorbike, so I use over 1500 [Shillings] just to come to Chulaimbo and back” (#34, M, 45, Chulaimbo/Busia, treatment completer)	Both	**High ** Total: 195 Completer: 52 Non-Completer: 74 Non-Starter: 69
Poverty	“My parents said let us sell this cow for you to at least get one injection…I told them not to because I was wondering what will happen to my younger siblings… I felt like I will leave them in poverty” (#86, M, 36, MTRH, treatment non-starter)	Both	**Medium ** Total: 70 Completer: 13 Non-Completer: 6 Non-Starter: 51
Food Insecurity	“I think just those ones, planning for visiting the hospital also, you have to plan, like there is no money in the house then you are telling people that you are going to hospital, where is the money? There is no food, food is not there, you want to attend the clinics, it's a challenge, aah” (#34, M, 45, Chulaimbo/Busia, treatment completer)	Both	**Low** Total: 25 Completer: 6 Non-Completer: 5 Non-Starter: 14
Lack of Disability Insurance	“I was employed …You know every company has its own regulations, they give you specific period of time to see if you will recover, then they keep adding but if you are not recovering you are retired on medical grounds, yeah…because if you are not able to do the job, then they is no need for them to maintain you, that is what happened.” (#44, M, 46, Busia, treatment completer)	Both	**Low** Total: 2 Completer: 2 Non-Completer: 0 Non-Starter: 0
High Cost of Chemotherapy	“No, not because I had cancer, the money that was needed for my treatment was a lot, and they wondered what I would sell for my treatment.” (#54, M, 40, Busia, treatment non-completer)	Both	**Medium** Total: 80 Completer: 4 Non-Completer: 15 Non-Starter: 61
Lack of National Health Insurance Fund	“Whenever I came, the doctor would ask if I enrolled into NHIF, I told them I hadn’t, they would just write it down. I kept wondering what NHIF was…If I had tried it earlier on, I would have recovered by now.” (#79, F, 53, Kitale, treatment non-starter)	Both	**High** Total: 118 Completer: 28 Non-Completer: 40 Non-Starter: 50
**Structural**—Community			
Facilitators			
Community health worker	“Nyamrerwa (community health workers in his mother tongue), those people, they are supposed to come the way we are seated and teach you on this and that, but there are those whom you have to go to their houses. So, it is required that they also visit every village and know that who and who is sick and maybe someone is sick but fears to go to the hospital.” (#43, M, 40, Chulaimbo, treatment completer)	Both	**Low** Total: 30 Completer: 12 Non-Completer: 6 Non-Starter: 12
Other members in community with KS	“My sister brought in some guy who had had the sarcoma, exactly the same condition and him it was even worse because the two legs were affected equally, then he had recovered…He said, no you just continue. In fact, I was planning to…, because they told me that chemotherapy is the one that clears my blood, then I was planning to abscond the other the other sessions, then he told me, no, don't do that, you go for them you are going to heal. I thought, actually if this guy healed and he was like this, he was describing exactly what I feel, then I when I got out of hospital I didn't want to wait, I just wanted to continue with my chemo.” (#34, M, 45, Chulaimbo/Busia, treatment completer)	Initiation	**Low** Total: 6 Completer: 4 Non-Completer: 2 Non-Starter: 0
Community social support	“Its only people from my church that raised 1000 shillings for transport the first time I came. Its them that raised the money.” (#35, M, 50, Chulaimbo, treatment completer)	Both	**Low** Total: 22 Completer: 13 Non-Completer: 9 Non-Starter: 0
Barriers			
Low knowledge of KS in community	“I didn’t, I didn’t because I just only knew somebody who has this elephantiasis and when this things started appearing on my skin, I thought that maybe it is that disease that is starting to appear then when I came here, they told me that this is different from that one I thought, that it was Kaposi’s.” (#62, M, 35, Chulaimbo, treatment non-completer)	Initiation	**Low** Total: 21 Completer: 4 Non-Completer: 0 Non-Starter: 17
Lack of community social support	“People just came the first time, but they didn’t come the second time. They just came to plan but did not show up on the day of the fund raiser.” (#33. M, 34, Chulaimbo/Busia, treatment completer)	Both	**Low** Total: 21 Completer: 3 Non-Completer: 5 Non-Starter: 13
Community stigma	“That can help, it can help since when many people are…when they see someone’s health has gone down, they think that since they ever heard that that person is positive, they take as if they stopped medication or they continued… having unprotected sex, people think of things like those, yes.” (#75, F, 38, MTRH, treatment non-starter)	Both	**Low** Total: 9 Completer: 3 Non-Completer: 2 Non-Starter: 4
**Structural**—Health Centers			
Facilitators			
Social Worker	“At Eldoret, I pleaded and told them [social workers] I have just acquired my NHIF card just help me instead of just going home without any treatment, so they helped me and told me next visit make sure you come with money or have that NHIF card.” (#79, F, 53, Kitale, treatment non-starter)	Both	**Low** Total: 5 Completer: 0 Non-Completer: 0 Non-Starter: 5
Free chemotherapy at health center	“This treatment is costly, people spend millions to get treatment, we don't have that kind of money, but since treatment has been offered for free, you have to go.” (#54, M, 40, Busia, treatment non-completer)	Both	**Low** Total: 6 Completer: 0 Non-Completer: 2 Non-Starter: 4
Barriers			
Difficulty in finding transportation to oncology centers	“Ok that one is a challenge to me since at times am told to come to the hospital even at a date like today and from where I come from, I don’t have transport to come with, so if I totally lack transport then I would not come,” (#69, M, 27, Chulaimbo, treatment non-completer)	Both	**Low** Total: 5 Completer: 1 Non-Completer: 3 Non-Starter: 1
Far distance to oncology centers	“What I would like to request is that you could open another branch …the patients will not face the challenges of transport” (#48, M, 34, Busia, treatment completer)	Both	**Low** Total: 16 Completer: 9 Non-Completer: 6 Non-Starter: 1
Limited treatment days/Inconvenient appointment times	“At times you come, and you don’t find the doctors…I did not find them, but I found a guy that told me to come the following Thursday when the doctors are around.” (#69, M, 27, Chulaimbo, treatment non-completer)	Adherance	**Low** Total: 4 Completer: 2 Non-Completer: 0 Non-Starter: 2
Difficulty with Navigation	“It was hard because I was going there, and I didn’t know anyone. I only knew the doctor that took the biopsy, that the only person I knew, we communicated on phone till I got there.” (#33, M, 34, Chulaimbo/Busia, treatment completer)	Both	**Low** Total: 5 Completer: 1 Non-Completer: 3 Non-Starter: 1
Referral Issues	“I did hear them saying that when you go to Referral you should first book an appointment, then come back and wait for the day to be treated and that you cannot just go and be treated the same day without an appointment.” (#63, F, 35, MTRH, treatment non-completer)	Initiation	**Low** Total: 4 Completer: 1 Non-Completer: 2 Non-Starter: 1
Long lines	“Ok I always know that here in referral it is a must that you queue the whole day and even go home without any doctor helping you.” (#75, F, 38, MTRH, treatment non-starter)	Both	**Low** Total: 29 Completer: 14 Non-Completer: 8 Non-Starter: 7
**Information**			
Facilitators			
Understands chemotherapy can improve/cure KS	“Yes, when I started treatment, there is a doctor that told me that if I have money, if I start treatment early it will clear since it was not bad, yeah, that encouraged me, it encouraged me to come to Chulaimbo as they had advised me, it made me work hard. Then because I know my children are young and they still have to study, I had to come so that I can go back to normal so that I can push for my children to have a normal life.” (#70, M, 39, Chulaimbo, treatment non-completer)	Both	**Low** Total: 11 Completer: 8 Non-Completer: 1 Non-Starter: 2
Understands / accepts side effects	“It was…, according to how I was feeling, it was just as a result of the chemo, because what I was being told at the beginning, I came to realize [the side effects] at the 4th cycle.” (#37, M, 40, Chulaimbo, treatment completer)	Both	**Low** Total: 14 Completer: 7 Non-Completer: 3 Non-Starter: 4
Understands / accepts chemo regimen/schedule	“For the first cycle when I get the injection, every month, I came twice, meaning that for the injection, I always took four months to come in, because every month twice; after two weeks, after two weeks, then after that they gave me a period of two months for taking blood for the test for that disease if it’s still continuing or it is not continuing. I went to the lab; they took the HB then they told me to come back for another treatment for GEMSA.” (#62, M, 35, Chulaimbo, treatment non-completer)	Both	**Low** Total: 44 Completer: 25 Non-Completer: 17 Non-Starter: 2
Knows where to obtain chemo	“There is a nurse at Bungoma who told me there is a clinic; AMPATH at Referral where I will be given injections and these things will get better, so she is the one who encouraged me.” (#48, M, 34, Busia, treatment completer)	Initiation	**Low** Total: 3 Completer: 3 Non-Completer: 0 Non-Starter: 0
Barriers			
Chemotherapy is deadly	*“Interviewer: Mmh…but in your own opinion, do you think that the medication led to early death?”* “Respondent: Yes, it quickened.” (#82, F, 55, MTRH, treatment non-starter)	Initiation	**Low** Total: 14 Completer: 5 Non-Completer: 1 Non-Starter: 8
Knowledge of chemotherapy side effects	“That these drugs, if you have already started them, they will turn your body black and will shave the whole of your hair with pain, the stomach will swell, you know what I hear from people, that the stomach will swell, your hair will fall of like someone who has shaved with a razorblade and walking will be a problem.” (#41, M, 38, Chulaimbo, treatment completer)	Both	**Low** Total: 24 Completer: 14 Non-Completer: 7 Non-Starter: 3
Does not know what chemotherapy is	“Oooh those drugs, I have never gotten time to understand what those drugs are…yeah now that I was sick and what I wanted was to receive treatment and get well so I have not gotten time to ask what they are maybe I will ask once am healed.” (#69, M, 27, Chulaimbo, treatment non-completer)	Initiation	**Medium** Total: 71 Completer: 35 Non-Completer: 22 Non-Starter: 14
Belief in traditional medicine	“There are better traditional medications compared to chemotherapy…they are more powerful, without side effects like chemo” (#42, M, 43, Busia, treatment completer)	Initiation	**Low** Total: 2 Completer: 1 Non-Completer: 5 Non-Starter:1
Cancer cannot be cured	“No! They didn't say anything, people were just shocked, cancer is shocking! Some said I should start medication, but then i said if it's Cancer I won't start because there's a wife to my brother, who was on treatment for cancer here, but she died, so I said to myself if it's the same as that of XXXX [sister in-law’s name], let me just die…yeah.” (#82, F, 55, MTRH, treatment non-starter)	Initiation	**Low** Total: 38 Completer: 17 Non-Completer: 15 Non-Starter: 6
**Motivation**—Interpersonal			
Facilitators			
With medical staff	“The doctors at one time helped me, like you came through for us one time we were stranded. The doctors have helped us on 2 occasions…They have been treating me like their fellow human being.” (#33, M, 34, Chulaimbo/Busia, treatment completer)	Both	**Low** Total: 7 Completer: 2 Non-Completer: 4 Non-Starter: 1
With social network	“I had faith that I would be okay, because by the second week the people who thought that I was dying came home and told me that I would be cured that the doctor who was treating me knew what he was doing.” (#54, M, 40, Busia, treatment non-completer)	Both	**Low** Total: 7 Completer: 5 Non-Completer: 2 Non-Starter: 0
Barriers			
Due to medical staff	“No there was none, there was none since it was not explained that I wait for 2 weeks then come back. That is the only thing that was not explained to us…no, when we got a different doctor, they told us a different thing then you come get another one who explains to you. For us to get lost is because all of them did not explain to us how it was…since today you could find one doctor and tomorrow you find a different one…eeh.” (#56, F, 57, MTRH treatment non-completer)	Both	**Low** Total: 8 Completer: 2 Non-Completer: 3 Non-Starter: 3
Due to social network	“Sometimes I call my dad and he tells me that I talk to my in-laws. When I try to talk to them [in-laws] they don’t respond.” (#85, F, 24, Kitale, treatment non-starter)	Both	**Low** Total: 10 Completer: 1 Non-Completer: 3 Non-Starter: 6
**Motivation**—Intrapersonal			
Facilitators			
Severity of KS symptoms motivation to be treated	“It was the suffering, and I was motivated…My body was in pain…That is what helped.” (#32, M, 20, MTRH, treatment completer)	Both	**Low** Total: 31 Completer: 16 Non-Completer: 14 Non-Starter: 1
Faith in hospital systems	“When I got to referral, I already had faith that I had been cured…Immediately I saw the hospital I just had faith that I had been cured.” (#58, F, 36 Webuye, treatment non-completer)		**Low** Total: 4 Completer: 1 Non-Completer: 2 Non-Starter: 0
For family/Wants to support family	“Yes, I wanted so much to be cured, I looked at my young children and I didn’t want to leave them before building their future.” (#53, M, 34, MTRH, treatment non-completer)	Both	**Low** Total: 11 Completer: 5 Non-Completer: 6 Non-Starter: 0
Barriers			
Poor health (cannot get to treatment facility)	“Yes, I had challenges getting time, when I came; they asked me why I was delaying in coming on the appointment dates? I told them it was hard to walk from my house to the road…When I tried to board the motorbike, I was unable to, I asked some women to help me, which they did. He brought me here (referring to Kitale AMPATH clinic), on alighting, thank God doctors were here, so they supported me off the bike and they expedited the process. When we finished, they helped me back on the same motorcycle, and I was taken back to my house, and I paid him 200 shillings. I told the doctors that I had taken long because of the illness.” (#79, F, 53, Kitale, treatment non-starter)	Both	**Low** Total: 9 Completer: 2 Non-Completer: 2 Non-Starter: 5
Lost hope	“You just die, the point I had gotten to! I just knew there was no more hope, I had started having bad thoughts because I had suffered a lot.” (#45, M, 31, Chulaimbo, treatment completer)	Both	**Low** Total: 14 Completer: 9 Non-Completer: 2 Non-Starter: 3
Fear of chemotherapy	“Ok what made me not to use chemotherapy, according to how I know chemotherapy, after one receives chemotherapy, it is only death there is no healing. So, I thought I was going to die and leave my children, it is better that I use ARVs the way I was told and maybe God will help me to get well and do my duties as normal.” (#75, F, 38, MTRH, treatment non-starter)	Initiation	**Low** Total: 11 Completer: 5 Non-Completer: 1 Non-Starter: 5
Lived side effects of chemotherapy	“It is just that blood, I became dark. And I had a lot of dots on the soles of my feet. Then at that time I lost my appetite seriously. I couldn’t eat. But I used to persevere because I wanted to get well, and I had to continue.” (#71, F, 33, Chulaimbo, treatment non-completer)	Adherence	**Medium** Total: 67 Completer: 37 Non-Completer: 30 Non-Starter: 0
Other co-morbid diseases	“Mmmh, no I had, I was sickly but not necessarily from the cancer, I had other conditions, mmmh, I was sickly, malaria, what, mmmh. Other conditions, mmmh, but I don't know if the cancer was also causing.” (#34, M, 45, Chulaimbo, Busia, treatment completer))	Both	**Low** Total: 4 Completer: 2 Non-Completer: 0 Non-Starter: 2
**Behavioral Skills**			
Facilitators			
Activation skills	“It was, because you do come early, the service providers come late, so we end up taking a lot of time maybe you are coming from far, you started early, even at times without any even breakfast, or maybe you would wish to take but you had only the transport, so you just come minus that. Reaching here you wait, you get hungry, but you just have to wait until you get your turn comes, is when you go back. So that long waiting is also…, is a challenge…it doesn't discourage me because it is my life. I am looking at my life, so whatever…, however challenges, I have to keep on, yes.” (#37, M, 40, Chulaimbo, treatment completer)	Both	**High** Total: 195 Completer: 120 Non-Completer: 37 Non-Starter: 38
Functional skills	“It’s only upon me to remind my supervisor that on a such a date I will be away on the oncology treatment.” (#37, M, 40, Chulaimbo, treatment completer) “I just have to know how much I need for example for transport, food, and medication maybe for other little expenses.” (#42, M, 43, Busia, treatment completer)	Both	**High** Total: 105 Completer: 61 Non-Completer: 35 Non-Starter: 9
System navigation skills: HIC /Oncology care separate, self- advocacy, referral system	“It’s aah, actually, first of all it's a short notice, then you know there is.., there other there other duties, there are other… what can I …, other calls like for example the HIV I have to go for where I am supposed to…, like I am supposed to be coming here [Busia] for my HIV clinics but I don't, and in Chulaimbo what they normally do they just give me the drugs they don't go into…, because they see that is a Busia patient, and then I have transferred from my local …, because of oncology, eeh so those are the challenges. So, the others, it’s like I have now concentrated more on this cancer than on the other on the other treatments.” (#34, M, 45, Chulaimbo/Busia, treatment completer)	Both	**Low** Total: 11 Completer: 11 Non-Completer: 0 Non-Starter: 0
Barriers			
Lack of activation skills	“They told me I delayed when I went there on 2nd, I took time to go back there, I was supposed to go on March, but I went there on 2nd May…I just waited because they had told me that they would call me by phone.” (#83, M, 32, Busia, treatment non-starter)	Both	**Low** Total: 10 Completer: 0 Non-Completer: 0 Non-Starter: 10
Lack of functional skills	“And now let us say when you are coming here for your treatment, how will you be planning for your transport?” **“Planning?”** “Yes, because you’ve said that you are thinking of starting, how will you be planning for your transport” **“God knows, I can’t say anything on that. I leave everything to God.”** (#84, F, 30, Chulaimbo, treatment non-starter)	Both	**Low** Total: 2 Completer: 1 Non-Completer: 0 Non-Starter: 1

Type of theme indicates whether the theme was for chemotherapy initiation, adherence, or both. Incidence density indicates whether the theme was high density (> 100 responses), medium density (50 to 100 responses) or low density (< 50 responses)

### Situated—economic/political/societal

There were multiple overarching situated barriers and facilitators which influenced chemotherapy initiation and adherence. One of the most common barriers was not having the financial resources to afford transportation to chemotherapy, chemotherapy medications, or food, with one patient reporting *“My parents said let us sell this cow for you to at least get one injection…I told them not to because I was wondering what will happen to my younger siblings… I felt like I will leave them in poverty.”* (#86, M, 36, MTRH, treatment non-starter) Transportation in particular was very costly for many patients, who often had to travel long distances to reach one of few centers providing chemotherapy.

A prominent facilitator to chemotherapy initiation and adherence was having the Kenyan government sponsored National Health Insurance Fund (NHIF) health insurance, which paid for the majority of patients’ treatment. A few patients also had work-sponsored sick leave or disability insurance to cover loss of income while receiving treatment. Additionally, some patients were part of microfinance groups, often either through HIV support groups or faith-based networks. Lastly, external funding sources such as through AMPATH or private donors who sponsored the cost of KS treatment was another notable facilitator. As one patient reported, *“My NHIF card helped me…When I went to Webuye, they first asked me if I had an NHIF card. I told them I had one. So whenever I came here, I would only incur cost of transport, but treatment and everything else was covered by NHIF.”* (#52, M, 34, Webuye, treatment completer) In particular, at the AMPATH center, Chulaimbo, all chemotherapy was provided free of charge for a period of time during this study through a donor sponsored program.

### Situated—community

At the community level some patients reported limited community social support and in fact reported a large amount of stigma after being diagnosed. Some patients believed this stigma was related to having visible skin lesions, while others believed the stigma was more related to the diseases processes itself including HIV and cancer. As one patient stated, *“In the village… someone could come to visit you at home, but his intentions are to spy on you, when they go away, you get to hear other things. You hear them say that so and so has bewitched you. They are not of any help; they come to visit you but are not of any help.”* (#43, M, 40, Chulaimbo, treatment completer) A few patients additionally reported that they were the first person in their community to be diagnosed with KS, which they felt limited community support and understanding of how to proceed with treatment. This was often a barrier specific to chemotherapy initiation rather than adherence.

In contrast, there were many themes relating to facilitators for starting and adhering to chemotherapy. Multiple patients found social support in their larger community, including meeting other patients in the community with KS or having community, often faith-based fund raisers for treatment. For example, one patient stated, *“The church too supported me…The also came to visit me and pray for me, if I told them I did not have transport to the hospital, they could contribute and give me, and they also encouraged me that I should go to hospital.”* (#63, F, 35, Chulaimbo, treatment non-completer) Some patients also found community health workers who would monitor their HIV care an important source of advice and support.

### Situated—health centers/system

Within the health care system, many larger barriers and facilitators influenced a patient’s access to chemotherapy initiation and adherence. Prominent barriers within the health system were relatively limited sites from which patients could obtain chemotherapy, which led to issues with transportation. Furthermore, many sites only offered chemotherapy every week or every other week on a specific day, leading to frequent scheduling conflicts, as one patient described *“I wasn't happy at all… I am a widow, I have children and I don’t have anyone supporting me, and my appointments are very close, after every two weeks. Looking for that transport is very hard. So, it can discourage you and you can stop the medication.”* (#71, F, 33, Chulaimbo, treatment non-completer).

At the larger tertiary hospital in particular, some patients found it difficult to navigate among different specialist services, such as from HIV care to oncology care. When patients did manage to navigate to the oncology center, they regularly had to wait for many hours to see a provider and oftentimes there were additional delays for scheduling the chemotherapy start date.

Facilitators to chemotherapy initiation and adherence included an easy chemotherapy referral process and free chemotherapy. Furthermore, at the large teaching and referral hospital there were social workers available to help with costs of chemotherapy and help patients enroll in NHIF, allowing their chemotherapy costs to be covered by insurance.

### Information

In addition to the larger structural barriers and facilitators identified, within the ecologic framework there were also multiple individual level themes related to chemotherapy adherence and initiation. The first related to patient’s knowledge of KS and its treatment modalities. Prominent barriers were limited knowledge or false beliefs, including that chemotherapy is deadly, that cancer cannot be cured, and not understanding the purpose of chemotherapy. As one patient stated, *“I just know that when you receive chemo, it is death and there is no healing.”* (#75, F, 38, MTRH, treatment non-starter).

An additional barrier was the knowledge of chemotherapy side effects—some of which were accurate, and others were likely over-estimations of the negative consequences of chemotherapy. An additional barrier was the use of traditional medicine instead of chemotherapy.

Facilitators were patients understanding the positive impacts of chemotherapy to improve or cure KS, understanding the chemotherapy regimen and timing of doses, and having knowledge about health centers where chemotherapy was available. An additional facilitator was when patients were aware of which chemotherapy side effects to expect, and then were able to tolerate these side effects to continue to return for further rounds of chemotherapy.

### Motivation

Patients additionally described multiple interpersonal and intrapersonal motivations related to chemotherapy initiation and adherence. Barriers included having multiple different healthcare providers with inconsistent information, lack of appropriate provider follow up, lack of education from providers about chemotherapy schedule and side effects, general mistrust of the healthcare system, and negative interactions with healthcare workers such as being reprimanded by providers. For example, one patient described *“That made me anxious about going to the hospital…because of your status [HIV status], they will not speak to you well… that would make you want to give up.”* (#78, M, 35, Kitale, treatment non-starter) Negative motivators from family and friends were stigma surrounding chemotherapy and a cancer diagnosis, as well as lack of support for pursuing KS treatment.

Interpersonal motivation often occurred between the patients and their providers as well as between the patients and their support network. Motivators for chemotherapy initiation and adherence included positive experiences with providers, consistent and accurate provider information, positive experiences with research staff, and both emotional as well as material support from family and friends.

Patients additionally described intrapersonal motivation barriers and facilitators. Intrapersonal barriers to chemotherapy were having poor health, losing hope, being fearful of chemotherapy, experiencing side effects of chemotherapy, and having co-morbid disease processes take priority (ex. Managing HIV care or pregnancy care). Many participants who reported being fearful or the chemotherapy or associated side effects noted this to be a large barrier to chemotherapy initiation.

Facilitator themes were motivation to seek or continue treatment from their severity of KS, faith in the healthcare system, and motivation to support their family. For example, a patient explained *“What helped me was the disease that I suffered from…It was smelling…It made me come for treatment.”* (#54, M, 40, Busia, treatment non-completer).

### Behavioral skills

Lastly, patients either acquired or lacked various behavioral skills which impacted their initiation and continuation of chemotherapy.

The activation skills which facilitated chemotherapy were coping skills, resilience, garnering social support, prioritizing oncology care, acceptance of disease, and accepting some of the hassles of the healthcare system such as long lines and wait times from treatment.

Functional skills which facilitated chemotherapy were setting and remembering chemotherapy appointment times, arranging leave for work for chemotherapy, arranging money for transportation and chemotherapy costs, and communicating concerns and wishes to their healthcare provider. As one patient with many functional skills described *“As soon as I clear one [appointment], I start looking for resources for the next [appointment]…So first of all, I make sure the NHIF card is active… and then I look for money for transport.”* (#34, M, 45, Chulaimbo/Busia, treatment completer) Some patients lacked these functional skills, which impeded their ability to initiate and adhere to chemotherapy. Themes included challenges planning for chemotherapy, arranging time off, arranging money, managing medications, and communicating with healthcare providers.

Lastly, system navigation skills that facilitated chemotherapy were a patient’s ability to navigate the logistics of chemotherapy and the complex referral system.

## Discussion

In this qualitative analysis, patients with HIV-associated KS in Kenya faced many barriers to initiation of and adherence to chemotherapy. The most significant barriers were lack of financial means, difficulty with convenience of appointments such as distance to facility, appointment times, long lines, limited appointments, intrapersonal barriers such as fear or hopelessness, and lack of proper or sufficient information about chemotherapy.

One of the common overall barriers to initiation and adherence was lack of financial means to afford not only chemotherapy but also transportation to appointments, and necessities such as food. Despite the fact that there are systems in place such as Kenya’s NHIF health insurance, which can often cover full chemotherapy treatment costs, and some external funding sources through AMPATH, patients with HIV-associated KS continue to face challenges with obtaining necessary funds. Prior studies in sub-Saharan Africa report that the path to obtaining health insurance (NHIF) can often be long and complicated with further limited access due to unaffordable monthly premiums [13, 14]. Financial insufficiency and poverty will likely continue to play large roles in the lives of people living with HIV-associated KS until these larger systems are able to implement initiatives that are both easy to access as well as maintain by all members of the population.

Despite local efforts to develop facilities and resources for providing care for KS, many patients still have to travel many hours to reach a care site, only to wait in line for extended periods of time. The infrequency and lack of consistency of appointments proved to be a major deterrent for patients to adhere to their chemotherapy regimen. In the future, resources should be directed towards expanding the number of trained medical personnel and equipment, increasing chemotherapy availability in terms of both location as well as appointment times, and creating a patient-friendly navigation system to avoid confusion and long lines [15, 16].

Lack of motivation and intrapersonal barriers such as fear and hopelessness played a large role in the delay or lack of chemotherapy initiation. The misconception that chemotherapy is associated with death and the fatalistic mindset that cancer is a death sentence were common during the interviews. Studies assessing the role of fear of disease and treatment in people living with HIV in SSA report the need for increased supportive and management counselling for patients and their support networks [17, 18]. The role of HIV, cancer, KS, and chemotherapy education also ties in closely with this barrier. Prior studies on HIV-associated malignancies in SSA note the need for education about oncological treatment and treatment benefits to improve treatment access and adherence [19, 20]. Educational interventions such as easy to understand pamphlets and public health campaigns should be expanded to alleviate feelings of fatalism, fear, and hopelessness.

Behavioral skills such as activation and functional skills played an important role in the initiation and adherence of chemotherapy regimens. Prior studies note that interventions to assist patients in acquiring, self-cueing, and self administering medications as well as an intervention to incorporate the treatment regimen into everyday life are important behvaioral skills to promote adherence [21, 22].

The findings in this study are essential to creating tailored interventions for this patient population. The multiple levels of the sIMB framework include national policy, community, health center, and individual targets. At the level of policy, our findings related to the difficulty accessing and maintaining NHIF may be relevant to ministry of health officials. At the level of community, community-wide education regarding KS and its treatment would enhance social support and potentially reduce KS-related stigma. At the level of health centers, the need for a navigation system and the importance of provider training are most applicable to improving the function of care programs. At the individual level, patient and caregiver education can enhance understanding, motivation, and support to initiate and complete chemotherapy.

The original IMB model created by Fisher and Fisher, was designed specifically for HIV prevention behaviors in the United States, and presented an individual focused rather than structural focused framework, which might be less applicable in most low resource settings [21]. Since this time, the original framework has been adapted to include frameworks such as sIMB and motivational interviewing based IMB (CLIMB), which address some of the gaps in the original framework [23–25]. These adapted IMB frameworks, specifically the sIMB model, better represented our patient interviews and provided a suitable framework for our study question.

A major limitation to this study was the population from which the patients for this study were recruited. This study population is a subset of a larger study population which was comprised of patients within AMPATH, who likely face fewer barriers to chemotherapy initiation and adherence than the majority of the population with HIV-associated KS.

## Conclusion

Interviews with KS patients in Kenya suggest the need to promote public health campaigns with reliable cancer and chemotherapy information, improve education about the chemotherapy process and side effects, increase oncology service ability, support enrollment in national health insurance, and increase incorporation of chronic disease care into existing HIV treatment networks. Furthermore, specific findings about layered HIV and cancer stigma highlight unique challenges with chemotherapy initiation and adherence for patients with HIV associated malignancies. Given the improved KS survival for patients on combination ART and chemotherapy, it is it is imperative to work on intervention implementation from the structural level to the intrapersonal level to ensure chemotherapy initiation and adherence.

## Data Availability

The datasets used and/or analysed during the current study are available from the corresponding author on reasonable request.

## Abbreviations

KS: Kaposi’s Sarcoma
SSA: Sub-Saharan Africa
ART: Antiretroviral Therapy
AMPATH: Academic Model Providing Access to Healthcare
MTRH: Moi Teaching and Referral Hospital
UGDH: Usain Gisu District Hospital
sIMB: Situated Information Motivation Behavior model
NHIF: National Health Insurance Fund
